# A Randomized Crossover Trial of Ivabradine, Propranolol, and Placebo in Postural Orthostatic Tachycardia Syndrome: A Detailed Description

**DOI:** 10.1016/j.jacadv.2026.102795

**Published:** 2026-05-13

**Authors:** Jaiden Uppal, Paras Deol, Priyanshu Giri, Agamjot Singh, Rasha Hamzeh, Jiyao Qi, Derek S. Chew, Mary Runte, Robert S. Sheldon, Satish R. Raj

**Affiliations:** a Department of Cardiac Sciences, Libin Cardiovascular Institute, Cumming School of Medicine, University of Calgary, Calgary, Alberta, Canada; b Vanderbilt Autonomic Dysfunction Center, Division of Clinical Pharmacology, Department of Medicine, Vanderbilt University Medical Center, Nashville, Tennessee, USA

**Keywords:** autonomic dysfunction, autonomic nervous system, clinical trial, medications, postural orthostatic tachycardia syndrome, treatment

## Abstract

**Background:**

Postural orthostatic tachycardia syndrome (POTS) is characterized by an excessive orthostatic heart rate increase and lacks approved pharmacologic therapies.

**Objectives:**

The objective of the study was to compare hemodynamic effects of ivabradine and propranolol and assess medication preferences in patients with POTS.

**Methods:**

In a randomized, placebo-controlled, crossover trial, patients with POTS completed three 4-week treatment phases (ivabradine, propranolol, and placebo). Each phase concluded with a 10-minute head-up tilt test with continuous beat-to-beat hemodynamic monitoring. Analyses included 28 participants (mean age 33 ± 9 years, 100% female). The prespecified primary outcome was delta heart rate during tilt. Head-up tilt test hemodynamics were described as minute-by-minute averages, deltas from supine baseline, and peak heart rate (HR_Peak_) in the last 5 to 10 minutes. Prespecified within-participant comparisons were performed between treatments. Treatment preference was recorded at each phase end.

**Results:**

Compared to placebo, HR_Peak_ was lower for ivabradine (99 ± 3 vs 118 ± 3 beats/min; *P* < 0.001) and propranolol (100 ± 3 vs 118 ± 3 beats/min; *P* < 0.001). ΔHR_Peak_ (standing – supine) was lower for ivabradine (27 ± 2 vs 36 ± 2 beats/min; *P* = 0.001) and propranolol (28 ± 2 vs 36 ± 2 beats/min; *P* = 0.003) vs placebo. Ivabradine showed a greater increase in Δ systolic blood pressure compared to propranolol (4.9 mm Hg vs 1.9 mm Hg; *P* = 0.001) but no significant difference in either HR_Peak_ or ΔHR_Peak_ between them. Among 22 preferences, all participants preferred ivabradine (*P* < 0.001) or propranolol (*P* < 0.001) over placebo, without differences between active treatments (59% vs 41%; *P* = 0.52).

**Conclusions:**

Ivabradine and propranolol reduce orthostatic tachycardia vs placebo, lowering heart rate below POTS diagnostic criteria. Ivabradine elevates systolic blood pressuremore than propranolol, supporting personalized drug selection. Importantly, heart rate lowering was consistent whereas symptom and quality-of-life effects were more selective, supporting individualized treatment.

Postural orthostatic tachycardia syndrome (POTS) is a disorder of the autonomic nervous system characterized by an exaggerated increase in heart rate (HR) within 10 minutes of standing, in the absence of orthostatic hypotension (blood pressure drop >20/10 mmHg).^1^, ^2^, ^3^ POTS predominantly impacts premenopausal (13-50 years) females and impacts up to 1% of the general population, a prevalence that is likely higher now.^3^ POTS is associated with a variety of chronic (>3 months), debilitating cardiovascular and neurological symptoms, which improve with recumbence.^3^ There is no specific cardinal symptom, or a critical number of symptoms needed for POTS diagnosis,^3^ although the orthostatic symptoms must be dominant in the clinical presentation and have been present for more than 3 months.^3^

Currently, there are no approved pharmacologic treatments for patients with POTS. Patients are treated with nonpharmacological interventions (eg, increased salt and water intake, compression garments, and exercise) and often prescribed a combination of off-label medications, such as ivabradine or propranolol.^3^ Ivabradine selectively blocks the I_f_ channels located in the sinoatrial node of the heart, lowering HR directly without impacting other systemic functions like blood pressure.^4^^,^^5^ Propranolol is a nonselective beta-blocker that has been shown to mitigate tachycardia and symptoms in patients with POTS, but may have off-target side effects due to the broad distribution of beta-adrenergic receptors throughout the body.^6^, ^7^, ^8^

A limited number of prior studies have examined the effects of propranolol or ivabradine on POTS.^4^^,^^6^^,^^9^, ^10^, ^11^, ^12^ However, no studies have directly compared these 2 therapies. Given the lack of comparative data between ivabradine and propranolol, we conducted a randomized, placebo-controlled, double-blinded, crossover trial of ivabradine, propranolol, and placebo to evaluate their relative hemodynamic and symptomatic effects.

## Methods

### Subjects

Patients referred to the Calgary Autonomic Research Clinic at the University of Calgary, between February 2021 and June 2025 were candidates for inclusion in this study. Participant eligibility included a confirmed diagnosis of POTS in accordance with POTS consensus statement criteria.^13^ All participants provided written, informed consent before their inclusion. This study was approved by the University of Calgary Ethics Board (REB19-1437). Data were stored in a Research Data Capture database.^14^ The data reported are a part of the “Crossover Study of Propranolol vs Ivabradine in POTS”. This project was completed in tandem with our qualitative interview study, aiming to understand the subjective patient experience of taking medications for POTS symptoms (REB24-0970). POTS was defined as an increase in HR (≥30 beats/min) within 10 minutes of upright posture, in the absence of orthostatic hypotension (blood pressure drop >20/10 mmHg), and chronic orthostatic symptoms (≥3 months). All potential participants were between 18 to 60 years of age, both male and female, and able and willing to provide informed consent. Specific exclusion criteria included a seated resting HR <60 beats/min in the absence of rate-lowering medications, supine blood pressure <90/60 mmHg, overt causes for postural tachycardia, that is acute dehydration, presence of underlying structural heart disease, coronary artery disease or prior myocardial infarction, history of tachyarrhythmias, pre-existing long QT interval, history of sick sinus syndrome or high degree atrioventricular block, presence of pacemaker, implantable cardioverter defibrillator, or cardiac resynchronization therapy device, diabetes mellitus, bronchospasms or uncontrolled asthma, pregnant or breast-feeding, inability to stop existing beta-blockers or ivabradine, concomitant use of class I and III antiarrhythmic agents or nondihydropyridine calcium-channel blockers, cytochrome P450 3A4 inhibitors, severe hepatic impairment, and any contraindication to propranolol or ivabradine. Participants that were given specific lifestyle instructions (in accordance with 2020 POTS Position Statement)^3^ before recruitment, including increased salt and water intake, compression garments, recumbent/upright exercise, were asked to keep these consistent during study duration. In addition, participants who underwent autonomic function testing before study inclusion were subtyped as hyperadrenergic or nonhyperadrenergic POTS based on upright norepinephrine levels (≥600 pg/mL).^3^

### Outcomes

The primary efficacy outcome of this study was the ΔHR during the head-up tilt test (HUT) after 4 weeks of treatment.

Secondary outcomes included absolute and delta hemodynamics during HUT, cognitive assessment via Cambridge Neuropsychological Test Automated Battery (CANTAB), quality-of-life/symptom surveys and questionaries, Holter monitor HR recordings, patient medication preferences, and qualitative patient interview responses.

### Study design

#### Crossover drug trial

**Figure 1:**
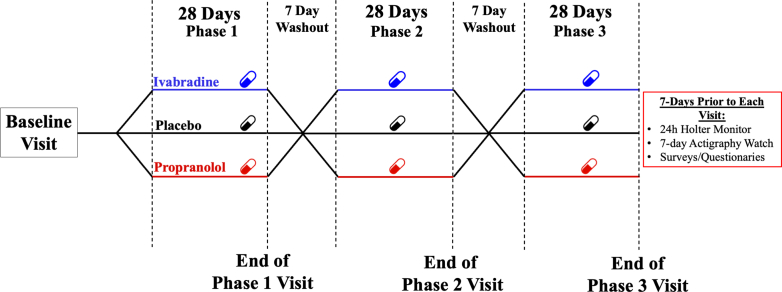
**Study Design Flowchart** Study design flowchart for the randomized crossover drug trial of ivabradine, propranolol, and placebo in POTS. Each phase is broken down into 28 ± 7 days with a 7-day washout period between phases.

Participants completed a double-blind, placebo-controlled, randomized crossover trial to compare effects of: 1) oral ivabradine 5 mg bid plus placebo (lactose) twice a day (to fill out four times a day schedule); 2) oral propranolol 10 mg four times a day (originally started at 20 mg 4 times a day [4 enrolled participants], then lowered to 10 mg four times a day in February 2022); and (3) oral placebo four times a day. Study pills were taken 4 time daily, usually taken with breakfast, lunch, dinner, and before bed. The order of all 3 interventions was randomized. After a baseline screening assessment (visit 1) following a washout period of 7 days, participants were to start with a 4-week course of ivabradine, propranolol, or placebo. The other 2 were given in separate 4-week courses with a 7-day washout period between phases (Figure 1).

#### Randomization

All participants received all 3 medications (ivabradine, propranolol, and placebo) in each phase of the study. The order of the interventions was randomized. Randomization was performed using randomization tables, by the Alberta Health Services (AHS) Research Pharmacy, which was not involved with participant recruitment or the study day and data collection.

#### Blinding

The study drugs (including the placebo) were purchased from the AHS Research Pharmacy and put into opaque digestible capsules (purchased from AHS research pharmacy) by personnel not involved with the conduct of the study. The capsules were in coded containers to aid in blinding. Participants and study personnel were blinded to the intervention. Participants were unblinded at the end of the study.

#### Hemodynamic testing protocol

All participants were instrumented with a 5-lead electrocardiogram (IVY Biomedical Model 450C) and noninvasive beat-to-beat blood pressure finger cuff (BMEYE and Finapres NOVA) placed on the contralateral arm to the brachial arm blood pressure cuff. This recorded continuous HR, systolic blood pressure (SBP), and diastolic blood pressure (DBP), and mean arterial blood pressures. The finger cuff was placed on the third finger (middle finger). Finger blood pressure readings were verified using intermittent brachial blood pressure measurements throughout the study. The beat-to-beat blood pressure waveform was analyzed to obtain estimates of stroke volume (SV), cardiac output (CO), and systemic vascular resistance (SVR) using Modelflow waveform analysis.^15^ We calculated CO as the product of SV and HR. We calculated SVR as the mean arterial blood pressure divided by CO. This Modelflow analysis technique was validated against a continuous echocardiographic approach to provide estimates of advanced hemodynamics during orthostatic challenges.^16^ Analog signals were digitally sampled (500 Hz) and stored for offline analysis using custom written software in MATLAB r2024b (Mathworks).

Participants then completed neuropsychological testing to assess cognitive domains of memory, attention, and executive function using CANTAB.^17^

Following CANTAB, participants completed a 10-minute supine baseline before a 10-minute HUT. The tilt-table was tilted from 0 degrees to 80 degrees head-up over 15 seconds. We used 80 degrees as literature has noted that the tilt angle should be 60 to 80 degrees to obtain maximal gravitational stress while minimizing skeletal muscle contraction.^18^^,^^19^ Although supine, brachial arm cuff measurements were taken at the start and end of the 10-minute baseline. Once tilted, brachial arm cuff measurements were conducted at minutes 1, 3, 5, and 10. Participants were asked about their symptoms at each minute while upright. Throughout each test, hemodynamics was continuously recorded, and symptoms were assessed during the baseline and at the end of the HUT using the Vanderbilt Orthostatic Symptom Score (VOSS).^20^ The VOSS questionnaire collected symptom severity of 9 common orthostatic symptoms on a scale of 0 to 10, with 10 being the worst. These included: mental clouding; blurred vision; shortness of breath; rapid heartbeat; tremulousness; chest discomfort; headache; lightheadedness; and nausea. The sum of the individual components provided a total score out of 90. This provided us with a baseline score, a post-HUT score, and a delta (Δ) VOSS score.

#### Cognitive testing

Seated cognitive testing (∼55 min) was performed using CANTAB. Tests included assessments of reaction time (psychomotor speed), rapid visual information processing (attention), paired associates learning (visual episodic memory), spatial span (working memory), verbal recognition memory (verbal memory), multitasking test (response inhibition), stockings of Cambridge (planning), and the emotional bias task (emotion processing). Tasks were administered on a touchscreen tablet with standardized instructions and built-in practice trials to minimize learning effects. The rapid visual information processing test was repeated in the standing position (∼10 min). These categories were compared between different drug phases.

#### Final study visit

At the end of the final visit, participants were asked which phase they preferred. This was implemented later during the study, with 22 participants providing preference data. Participants and study team were then unblinded.

#### Quality-of-life and symptom surveys/questionnaires

One week before each study visit, participants completed health-related quality-of-life (QoL) surveys/questionnaires. The surveys included RAND-36,^21^ a 36-item questionnaire that measures overall health-related QoL across eight domains: physical functioning, pain, emotional well-being, and social functioning. COMPASS-31^22^ is a 31-item questionnaire quantifying autonomic symptom severity across six domains: orthostatic intolerance, vasomotor, secretomotor, gastrointestinal, bladder, and pupillomotor function. Orthostatic Grading Scale^23^ is a questionnaire assessing the severity of orthostatic intolerance symptoms and their impact on daily activities. Malmo POTS Symptom Score (MAPS)^24^ is a standardized questionnaire evaluating the frequency and severity of POTS symptoms; lightheadedness, palpitations, fatigue, and difficulty concentrating. This was implemented late, with 14 participants completing this survey. anxiety visual analog scale (VAS)^25^ is a self-report tool where individuals rate their current level of anxiety on a continuous line. Once survey scores were obtained, we analyzed category and total scores between the drug phases. Surveys were distributed and administered to participants via Research Data Capture.

#### Holter monitor recordings

Participants received a Bittium Faros Holter monitor (Bittium Corporation) to record a 24-hour electrocardiogram for changes in HR. This was worn immediately after the baseline visit (visit 1) for 24 hours. This was connected again by the participant roughly 1-week before the end of each cycle, for 24 hours.

#### Qualitative interview project

Participants who completed the crossover drug trial were invited to conduct a qualitative interview (n = 11). They were asked whether they preferred any study medications, and to describe reasons underlying their preference, including symptoms, side effects, and overall tolerability. Interviews were recorded and transcribed for thematic analysis. This study was a separate protocol and approved by the University of Calgary ethics board (REB24-0970).

### Data analysis

Supine hemodynamics was calculated as the mean values across the full 10-minute supine preceding HUT. Upright minute-by-minute averages (for each of the 10-minutes) were calculated for each test using the 60 seconds in each minute. Minute-by-minute hemodynamic deltas (Δ) were calculated as the minute averages in the upright position minus the supine average. The peak 1-minute average HR (HR_Peak_) and delta (ΔHR_Peak_) between minutes 5 and 10 was also obtained. To assess the therapeutic efficacy of the drugs, we used the orthostatic index (OI) formulation that reflects both prolongation of tilted time and suppression of tachycardia.^26^ The OI is the area under the curve of RR interval (in seconds/beat) over the HUT test (maximum of 10 minutes). Greater OI indicates improved orthostatic tolerance with longer upright time and/or reduced upright HR.

The 24-hour Holter monitor recordings were divided into day (7 am to 10 pm), night (10 pm to 7 am), and 24-hour. Each period consisted of a mean HR, instantaneous maximum HR, and instantaneous minimum HR. HRs were compared between-drug phases. Data were analyzed using custom written software in MATLAB r2024b (Mathworks).

#### Statistical analysis

The primary outcome was the ΔHR during the HUT after 4 weeks of treatment. Normality was assessed via Shapiro-Wilks test. Data are represented as mean ± SEM unless indicated otherwise. Continuous variables were compared via linear mixed model^27^ and represented using estimated marginal mean values. This was run as a single model, with pairwise Bonferroni correction. *P* values from the linear mixed model are denoted as P_Time_, P_Drug_, and P_Int_. P_Time_ demonstrates the comparison of the hemodynamics across each minute during the 10-minute HUT. P_Drug_ demonstrates the comparison of ivabradine vs placebo, propranolol vs placebo, and ivabradine vs propranolol. P_Int_ tested interaction effects, whether the effect of the drugs varied across time points, specifically, whether the trajectory of change over time differed between treatment conditions. VOSS, health-related QoL surveys, peak HR (HR_Peak_), CANTAB, and Holter monitor were compared via Wilcoxon sign-rank tests. Participant drug preferences were compared using Cochranes Q and McNemar’s tests (n, %). HR assessment comparing hyperadrenergic and nonhyperadrenergic POTS were compared via Mann-Whitney U tests. A 2-tailed *P* value of <0.05 was deemed statistically significant. Statistical analyses were performed using SPSS statistical software for Windows version 29 (SPSS, Inc., IBM). Figures were created using GraphPad Prism (version 8.0.0 for Windows, GraphPad Software).

An intention-to-treat analysis was not performed because completion of the HUT protocol was required for outcome data. Participants who withdrew before completing the tilt protocol were excluded from analysis due to absence of outcome measurements.

#### Sample size calculation

We completed a 3-group sample size calculation. Assuming an intragroup HR variability (SD) of 15 beats/min, 20 participants would be required to detect a HR reduction (effect size) of 10 beats/min at 80% power and an alpha of 0.05. Sample size calculation was performed using Stata/IC 15.1. To allow for dropout and incomplete data collection, we aimed to enroll 45 participants, where we ended with 28 participants completed.

## Results

A concurrent Brief Report in JACC reports on the 3 pairs of between-drug comparisons for ΔHR, HR_Peak_, ΔHR_Peak_, 24-hour Holter mean HR, Orthostatic Grading Scale, RAND-36, and COMPASS-31 data.

### Demographics

**Figure 2:**
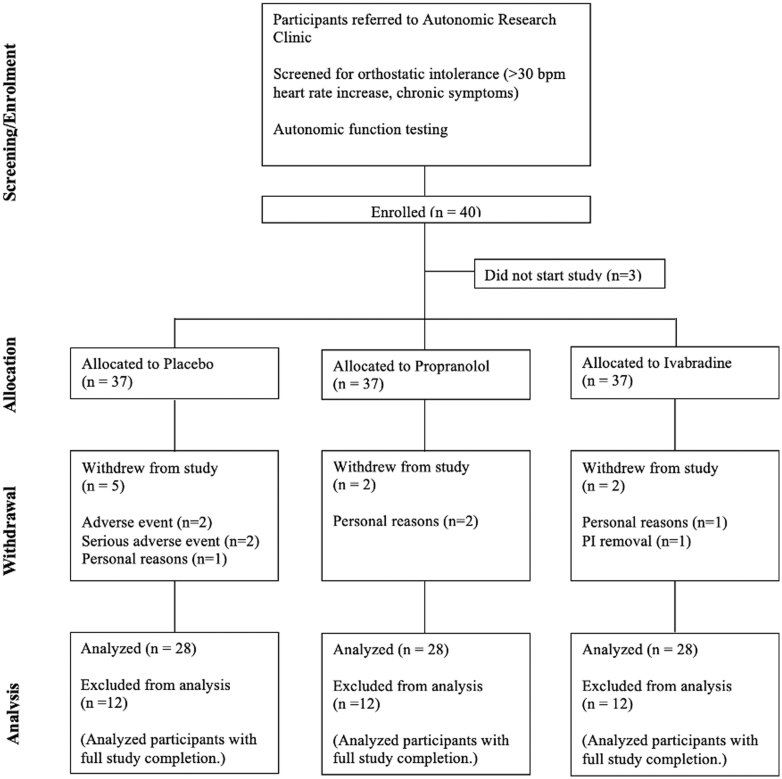
**CONSORT Participant Flowchart** Participant flowchart highlighting screening process, total enrolled (n = 40), withdrawals per drug phase, and total analyzed (n = 28). Specific category of withdrawal is included in the withdrawal sections. PI = primary investigator.

Twenty-eight participants with POTS completed the study (females, n = 28; age 33 ± 9 years) (Figure 2). Participant demographics are provided in Table 1. Withdrawal data are provided in Table 2. Most withdrawals (n = 8 [67%]) were during the placebo phase due to nonmanageable symptoms or personal/social reasons. No patients withdrew from the study due to the active study drugs (ivabradine or propranolol). Key results are summarized in the concurrently published *JACC* Brief Report. This article provides the full methodological, architectural, and operational detail supporting those findings.

**Table 1:** Participant Demographics

Demographics (n = 28)	
Age (years)	33 ± 9
Female (n)	28
Height (cm)	169 ± 8
Weight (kg)	68 ± 13
BMI (kg/m^2^)	24 ± 5.3
Hyperadrenergic POTS	N = 13/21
Baseline visit hemodynamics	
Supine	
HR (beats/min)	85 ± 18
SBP (mm Hg)	120 ± 17
DBP (mm Hg)	77 ± 14
CO (L/min)	6.0 ± 1.6
SV (mL)	73 ± 18
SVR (dyn·s·cm^−5^)	1,209 ± 646
10-min tilt	
HR (beats/min)	119 ± 18
SBP (mm Hg)	126 ± 18
DBP (mm Hg)	95 ± 14
CO (L/min)	5.2 ± 1.7
SV (mL)	50 ± 19
SVR (dyn·s·cm^−5^)	1,543 ± 670
Tilt peak HR (beats/min)	119 ± 18
Delta (tilt-supine)	
ΔHR (beats/min)	35 ± 12
ΔSBP (mm Hg)	5 ± 15
ΔDBP (mm Hg)	18 ± 12
ΔCO (L/Min)	−0.9 ± 1.0
ΔSV (mL)	−30 ± 15
ΔSVR (dyn·s·cm^-5^)	399 ± 414
ΔPeak HR (beats/min)	35 ± 12
Duration since POTS symptom onset	502 ± 762
Duration of POTS diagnosis	101 ± 153 d
Concomitant medications	
Ivabradine	Prohibited
Beta-blockers (propranolol, bisoprolol, metoprolol, carvedilol, nadolol)	Prohibited
Midodrine	Prohibited
Fludrocortisone	Prohibited
Pyridostigmine	Prohibited
Cytochrome P450 3A4 inhibitors (clarithromycin, erythromycin, diltiazem, itraconazole, ketoconazole, ritonavir, verapamil)	Prohibited
Cytochrome P450 3A4 inducers (phenobarbital, phenytoin, rifampicin, St. John’s Wort and glucocorticoids)	Prohibited
Class I antiarrhythmic agents (lidocaine, procainamide, propafenone)	Prohibited
Class II antiarrhythmic agents (amiodarone, dronedarone, sotalol, ibutilide)	Prohibited
Nondihydropyridine calcium channel blockers	Prohibited
All other medications	Maintained at a stable dosage throughout study duration
Relevant comorbidities (n)	
Migraines	11
Attention deficit hyperactivity disorder (ADHD)	8
Anxiety disorders	6
Ehlers-Danlos syndrome (EDS)	6
Depression	4
Asthma	4
Long-COVID	2
Fibromyalgia	2
Gastroesophageal reflux disease (GERD)	2
Joint hypermobility	1
Arthritis	1
Irritable bowel syndrome	1
Chronic fatigue syndrome (CFS)	1

Values are mean ± SD or n.

BMI = body mass index; CO = cardiac output; DBP = diastolic blood pressure; HR = heart rate; POTS = postural orthostatic tachycardia syndrome; SBP = systolic blood pressure; SV = stroke volume; SVR = systemic vascular resistance.

**Table 2:** Participant Withdrawal Breakdown

Record ID	Reason for Withdrawal	Phase of Withdrawal (1,2,3, if Applicable)	Drug of Withdrawal (if Applicable)	Previous Drug Phases
1	Did not complete baseline visit	NA	NA	NA
5	Personal reasons	2	Placebo	P1: IVA P2: Placebo
6	Withdrew due to SAE	1	Placebo	P1: Placebo
8	Patient was hospitalized for Psychiatric reasons and could not take her study drugs	2	Ivabradine	P1: Placebo P2: IVA
9	Patient got COVID-19 while in the study, which made her HR with the study drug go very low, so she opted out of the study	1	Propranolol	P1: Propranolol
10	Did not complete baseline visit	NA	NA	NA
15	Patient was removed from the study as she removed the drug encapsulation and was unblinded	2	NA	NA
23	Did not complete baseline visit.	NA	NA	NA
24	Could not tolerate cycle 1 drugs, decided it was best for her to discontinue with the study	1	Placebo	P1: Placebo
26	Patient experienced significant GI problems. After speaking with her family doctor, they both believe this is not related to the study medication as this has happened to her before. She could not hold down any medications or fluids without vomiting shortly after. She asked to withdraw given these symptoms.	1	Propranolol	P1: Propranolol
39	Withdrew due to SAE	3	Placebo	P1: Propranolol P2: IVA P3: Placebo
40	Patient experienced side effects and decided it was not a good time for her to continue with the study	1	Placebo	P1: Placebo

Participant withdrawal data with reason of withdrawal, phase of withdrawal, unblinded intervention, and previous phase interventions. Three participants did not start the study 4 withdrew due to personal reasons, 2 withdrew due to adverse events, 2 withdrew due to serious adverse events, and 1 withdrawal due to PI removal. There were a total of 12 withdrawals.

GI = gastrointestinal; IVA= ivabradine; SAE = serious adverse event; other abbreviation as in Table 1.

### Ivabradine vs placebo

#### Hemodynamic response to HUT

**Figure 3:**
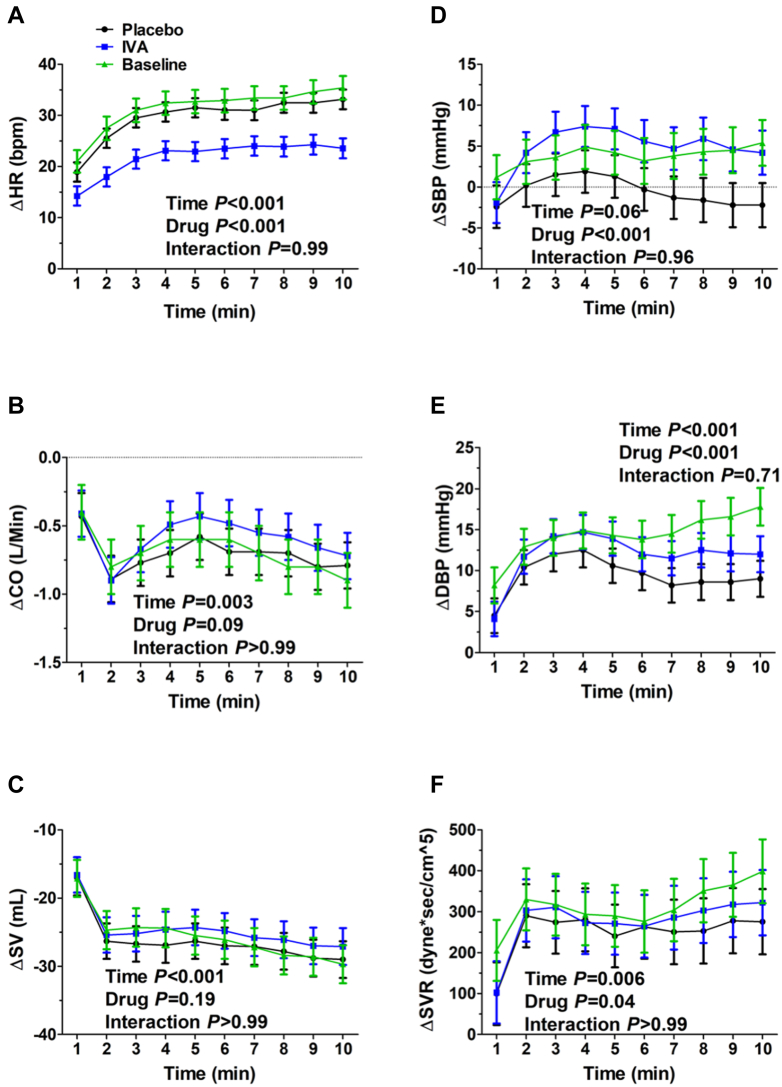
**Delta Hemodynamics (Ivabradine vs Placebo) Delta Hemodynamics During full 10-Minutes of Head-Up-Tilt for Ivabradine, Placebo, and Visit 1 (Baseline)** Estimated marginal mean data for (A) delta heart rate, (B) cardiac output, (C) stroke volume, (D) systolic blood pressure, (E) diastolic blood pressure, and (F) systemic vascular resistance are shown across the full 10-minutes of HUT for ivabradine (blue), placebo (black), and baseline visit (green). Data are shown for 28 participants. Data are represented as mean ± SEM. Numbers indicate significance level for time, ivabradine vs placebo (drug), and interaction from a linear mixed model. Less than *P* = 0.05 was considered significant. HR = heart rate; IVA = ivabradine; SBP = systolic blood pressure; DBP = diastolic blood pressure; CO = cardiac output; SV = stroke volume; SVR = systemic vascular resistance.

ΔHR increased significantly over time (P_Time_ < 0.001) (Figure 3A). ΔCO exhibited a time-dependent decrease (P_Time_ = 0.003) (Figure 3B). ΔSV exhibited a time-dependent decrease (P_Time_ < 0.001) (Figure 3C). ΔSBP experienced an increase with ivabradine (−0.3 mm Hg vs 5.1 mm Hg; P_Drug_ < 0.001) (Figure 3D). ΔDBP increased over time (P_Time_ < 0.001) and was larger in magnitude for ivabradine (9.4 mm Hg vs 11.9 mm Hg; P_Drug_ < 0.001) (Figure 3E). Lastly, ΔSVR increased significantly over time (P_Time_ = 0.006) and was larger in magnitude for ivabradine (247 dyn·s·cm^-5^ vs 267 dyn·s·cm^-5^; P_Drug_ = 0.04) (Figure 3F). None of the delta hemodynamics displayed interaction effects. There was a greater OI with ivabradine (5.2 ± 0.5 vs 4.2 ± 0.5; *P* < 0.001) compared to placebo. Absolute minute-by-minute data for each drug are provided in Supplemental Figure 1.

#### Peak HR hemodynamics during HUT

Aside from HR_Peak_ and ΔHR_Peak_, there were no other differences in absolute or delta hemodynamics at HR_Peak_.

#### 24-hour Bittium Holter monitor

The day and night mean HR was lower for ivabradine compared to placebo (Table 3). The maximum HR recorded was also lower for ivabradine for day and night (Table 3).

**Table 3:** 24-Hour Holter Monitor Recordings

					*P* Value		
	Time Period	Ivabradine	Propranolol	Placebo	IVA vs Placebo	Propranolol vs Placebo	IVA vs Propranolol
Mean HR	24-h	84 ± 19	86 ± 19	100 ± 18	**<0.001**	**<0.001**	>0.99
	Day	86 ± 17	89 ± 17	102 ± 17	**<0.001**	**<0.001**	>0.99
	Night	75 ± 24	78 ± 24	92 ± 23	**0.001**	**0.01**	>0.99
Maximum HR	24-h	157 ± 25	167 ± 25	178 ± 23	**0.004**	0.26	0.35
	Day	157 ± 25	167 ± 24	177 ± 23	**0.005**	0.27	0.39
	Night	94 ± 36	100 ± 35	123 ± 33	**0.005**	**0.03**	>0.99
Minimum HR	24-h	54 ± 9	54 ± 9	55 ± 8	>0.99	>0.99	>0.99
	Day	55 ± 10	55 ± 9	56 ± 9	>0.99	>0.99	>0.99
	Night	54 ± 9	54 ± 9	55 ± 8	>0.99	>0.99	>0.99

Values are mean ± SD unless otherwise indicated. Participant Holter monitor recording data over 24-h. Mean heart rate (HR), maximum HR, and minimum HR were recorded. Recoding was divided into 3-time windows; 24-h, day (7 am to 10 pm), and night (10 pm to 7 am). Data are presented at mean ± SD. Numbers indicate significance level for a paired comparison using a Wilcoxon sign-rank test. **Bold** indicates a *P*-value ≤ 0.05. This demonstrates a significant difference.

Abbreviation as in Table 2.

#### Participant-reported outcomes

There were no differences in VOSS total scores for both baseline (*P* = 0.6) and post-tilt (*P* = 0.09) (Table 4), but there was higher shortness of breath for placebo during baseline (1.6 ± 1.8 vs 0.8 ± 1.0; *P* = 0.01) and higher rapid heartbeat post-tilt (5.0 ± 2.7 vs 3.8 ± 2.9; *P* = 0.02) compared to ivabradine. There were no differences in ΔVOSS overall scores (*P* = 0.43) (Table 4).

**Table 4:** Quality-Of-Life and Symptom Surveys Scores

					*P* Value		
Survey	Subscale	Ivabradine	Propranolol	Placebo	IVA vs Placebo	Propranolol vs Placebo	IVA vs Propranolol
BSL VOSS	Mental clouding	2.0 ± 2.2	2.1 ± 1.9	2.5 ± 2.8	0.76	0.84	0.66
	Blurred vision	1.0 ± 1.5	1.3 ± 1.5	1.8 ± 2.0	0.06	0.16	0.24
	Shortness of breath	0.8 ± 1.0	1.3 ± 1.5	1.6 ± 1.8	**0.01**	0.57	**0.01**
	Rapid heartbeat	1.3 ± 1.4	1.4 ± 1.1	1.9 ± 1.9	0.28	0.36	0.33
	Tremulousness	0.7 ± 1.4	1.2 ± 2.0	1.5 ± 2.3	0.12	0.95	0.08
	Chest discomfort	1.2 ± 1.2	1.1 ± 1.6	1.4 ± 1.6	0.90	0.30	0.78
	Headache	2.9 ± 2.6	2.3 ± 2.5	2.3 ± 2.5	0.35	>0.99	0.15
	Lightheadedness	1.2 ± 1.5	1.4 ± 1.9	1.2 ± 2.1	0.67	0.61	0.53
	Nausea	1.0 ± 2.3	1.2 ± 2.5	1.6 ± 2.6	0.64	0.93	0.59
	Overall score	12.2 ± 11.9	13.4 ± 13.8	15.7 ± 15.7	0.6	0.39	0.32
Post-tilt VOSS	Mental clouding	4.0 ± 2.9	3.6 ± 2.9	4.9 ± 3.1	0.15	**0.005**	0.54
	Blurred vision	2.9 ± 2.7	2.9 ± 3.1	3.6 ± 3.1	0.09	0.17	0.76
	Shortness of breath	4.0 ± 2.9	4.2 ± 2.8	4.8 ± 3.0	0.11	0.24	0.69
	Rapid heartbeat	3.8 ± 2.9	3.8 ± 2.8	5.0 ± 2.7	**0.02**	**0.01**	0.93
	Tremulousness	3.0 ± 2.8	3.0 ± 3.1	3.9 ± 3.0	0.01	0.15	0.79
	Chest discomfort	3.3 ± 3.1	3.6 ± 2.8	4.0 ± 3.0	0.16	0.24	0.55
	Headache	3.7 ± 3.3	3.7 ± 3.3	3.9 ± 3.0	0.82	0.46	0.94
	Lightheadedness	4.6 ± 3.1	4.8 ± 3.2	5.2 ± 3.0	0.19	0.11	0.53
	Nausea	3.2 ± 3.4	2.8 ± 2.9	3.4 ± 3.3	0.52	0.15	0.77
	Overall score	32.5 ± 22.1	32.4 ± 22.8	38.8 ± 21.9	0.09	**0.04**	0.79
Delta VOSS	Mental clouding	2.4 ± 2.6	2.2 ± 2.4	2.4 ± 3.6	0.57	0.25	0.64
	Blurred vision	1.9 ± 2.0	2.2 ± 2.7	2.0 ± 3.1	0.44	0.98	0.64
	Shortness of breath	3.5 ± 2.8	3.7 ± 2.5	3.2 ± 3.2	0.75	0.33	0.90
	Rapid heartbeat	2.8 ± 2.5	3.3 ± 2.7	3.2 ± 3.4	0.19	0.84	0.43
	Tremulousness	2.5 ± 2.5	2.7 ± 2.2	2.3 ± 3.4	0.38	0.98	0.22
	Chest discomfort	2.4 ± 2.6	3.0 ± 2.4	2.5 ± 3.1	0.53	0.58	0.30
	Headache	1.2 ± 2.4	2.0 ± 2.1	1.6 ± 2.3	0.41	0.77	**0.02**
	Lightheadedness	3.5 ± 3.2	4.0 ± 2.7	3.9 ± 3.1	0.13	0.6	0.18
	Nausea	2.4 ± 3.4	1.9 ± 2.7	1.5 ± 3.3	0.94	0.71	0.9
	Overall score	22.5 ± 18.1	25.0 ± 17.0	22.5 ± 24.9	0.43	0.82	0.22
RAND-36	PF	42.1 ± 26.8	40.7 ± 23.8	37.7 ± 25.5	0.17	0.24	0.34
	RLPH	20.7 ± 37.6	21.6 ± 32.8	13.2 ± 29.7	0.16	0.25	0.16
	RLEH	48.3 ± 44.7	57.5 ± 38.8	48.3 ± 41.7	0.88	0.10	0.30
	EF	24.7 ± 22.8	26.0 ± 17.7	26.9 ± 19.0	0.60	0.93	0.67
	EWB	60.6 ± 24.8	63.4 ± 19.5	58.2 ± 21.4	0.41	**0.01**	0.34
	SF	47.0 ± 33.1	53.0 ± 26.4	49.3 ± 26.1	0.50	0.35	0.19
	Pain	55.7 ± 26.7	61.8 ± 26.5	49.7 ± 25.1	0.20	**0.006**	0.08
	GH	28.3 ± 18.2	29.8 ± 20.4	28.8 ± 20.1	0.56	0.56	0.32
	Physical health score	42.3 ± 25.1	44.3 ± 21.8	36.9 ± 23.7	0.10	**0.003**	0.57
	Mental health score	56.4 ± 31.1	61.5 ± 23.4	56.8 ± 24.5	0.87	0.07	0.34
COMPASS-31	Orthostatic intolerance	25.5 ± 8.5	25.5 ± 9.2	28.1 ± 6.9	**0.03**	0.07	0.37
	Vasomotor	2.8 ± 1.0	2.9 ± 1.0	3.0 ± 1.1	**0.007**	0.32	0.08
	Secretomotor	5.7 ± 4.2	4.9 ± 3.6	5.4 ± 4.0	0.52	0.24	0.12
	Gastrointestinal	11.2 ± 4.6	11.1 ± 4.7	11.4 ± 4.1	0.59	0.27	0.70
	Bladder	2.1 ± 2.2	2.1 ± 2.2	2.1 ± 2.4	0.66	0.79	0.60
	Pupillomotor	2.6 ± 1.1	2.4 ± 1.0	2.4 ± 1.0	0.34	0.58	0.10
	Total score	49.9 ± 17.1	48.8 ± 16.4	52.5 ± 14.8	0.13	**0.01**	0.98
OGS	Frequency of orthostatic symptoms	2.5 ± 1.1	2.6 ± 1.2	3.1 ± 0.8	**0.003**	**0.01**	0.61
	Severity of orthostatic symptoms	2.1 ± 0.8	2.2 ± 0.9	2.4 ± 0.7	**0.03**	0.33	0.42
	Conditions under which of orthostatic symptoms	2.8 ± 1.1	2.8 ± 1.1	3.1 ± 0.8	0.12	0.15	0.95
	Activities of daily living	2.1 ± 1.0	2.2 ± 0.9	2.3 ± 0.7	0.15	0.36	0.71
	Standing time	2.6 ± 1.2	2.6 ± 1.1	2.9 ± 1.1	0.09	0.16	0.98
	Total score	12.0 ± 4.4	12.3 ± 4.5	13.8 ± 3.5	**0.02**	0.08	0.58
MAPS	Chest pain	2.9 ± 2.7	3.2 ± 2.9	3.5 ± 3.2	0.36	0.37	0.64
	Headache	5.1 ± 2.5	5.0 ± 3.4	6.0 ± 2.7	0.26	0.18	0.68
	Difficulty concentrating	5.3 ± 2.4	5.4 ± 2.3	5.2 ± 2.6	0.64	0.96	0.83
	Muscle ache	4.8 ± 3.0	3.7 ± 3.0	4.7 ± 3.0	0.73	**0.01**	0.07
	Nausea	5.1 ± 3.3	5.1 ± 3.7	4.8 ± 3.6	0.88	0.72	0.94
	Gastrointestinal symptoms	4.7 ± 3.1	5.1 ± 3.7	5.2 ± 3.8	0.54	0.83	0.58
	Fatigue	7.1 ± 2.5	7.1 ± 2.3	7.3 ± 2.3	0.72	0.47	0.92
	Lightheadedness	4.8 ± 2.5	5.6 ± 2.1	5.5 ± 2.3	0.19	0.69	0.29
	Sense that you might faint	3.2 ± 2.0	4.6 ± 2.6	4.6 ± 2.7	0.053	0.44	0.12
	Palpitations	4.9 ± 2.7	4.5 ± 2.5	5.6 ± 2.2	0.72	0.32	0.58
	Shortness of breath	3.9 ± 2.2	4.2 ± 3.2	4.5 ± 3.4	0.35	0.32	0.61
	Sleep disturbances	5.1 ± 3.3	5.0 ± 3.5	5.5 ± 3.2	0.75	0.26	0.83
	Total score	57.4 ± 21.9	57.6 ± 27.9	61.0 ± 24.0	0.72	0.51	0.88
Anxiety VAS	Total score	55.3 ± 26.3	54.6 ± 26.0	53.5 ± 24.5	0.61	0.88	0.64

Values are mean ± SD unless otherwise indicated. Participant quality-of-life and symptom surveys scores. Patients were sent these surveys 1 week before eaach study visit. The Vanderbilt Orthostatic Symptom Score (VOSS) was used to collect symptom severity based on 9 common orthostatic symptoms. Symptoms are rated 0 (no symptoms) to 10 (worst symptoms). Overall score has a maximum score of 90. Delta VOSS was determined as the post-tilt score minus pretilt score. RAND-36 is a 36-item questionnaire that measures health-related quality-of-life across 8 domains. COMPASS-31 is a 31-item questionnaire that quantifies autonomic symptom severity across 6 domains. Orthostatic Grading Scale assess severity of orthostatic intolerance symptoms and impact on daily activities. Malmo POTS Symptom Score (MAPS) evaluates frequency and severity of POTS symptoms. Anxiety VAS is a self-report tool used to rate anxiety levels. Data are presented at mean ± SD. Numbers indicate significance level for a paired comparison using a Wilcoxon sign-rank test. **Bold** indicates a *P*-value ≤ 0.05. This demonstrates a significant difference.

BSL = baseline; EF = energy/fatigue; EWB = emotional well-being; GH = general health; OGS = Orthostatic Grading Scale; PF = physical functioning; RLEH = role limitation due to emotional health; RLPH = role limitation due to physical health; SF = social functioning; VAS = visual analog scale; other abbreviation as in Table 2.

There were no differences in MAPS and anxiety VAS scores between placebo and ivabradine (Table 4).

### Propranolol vs placebo

#### Hemodynamic response to HUT

**Figure 4:**
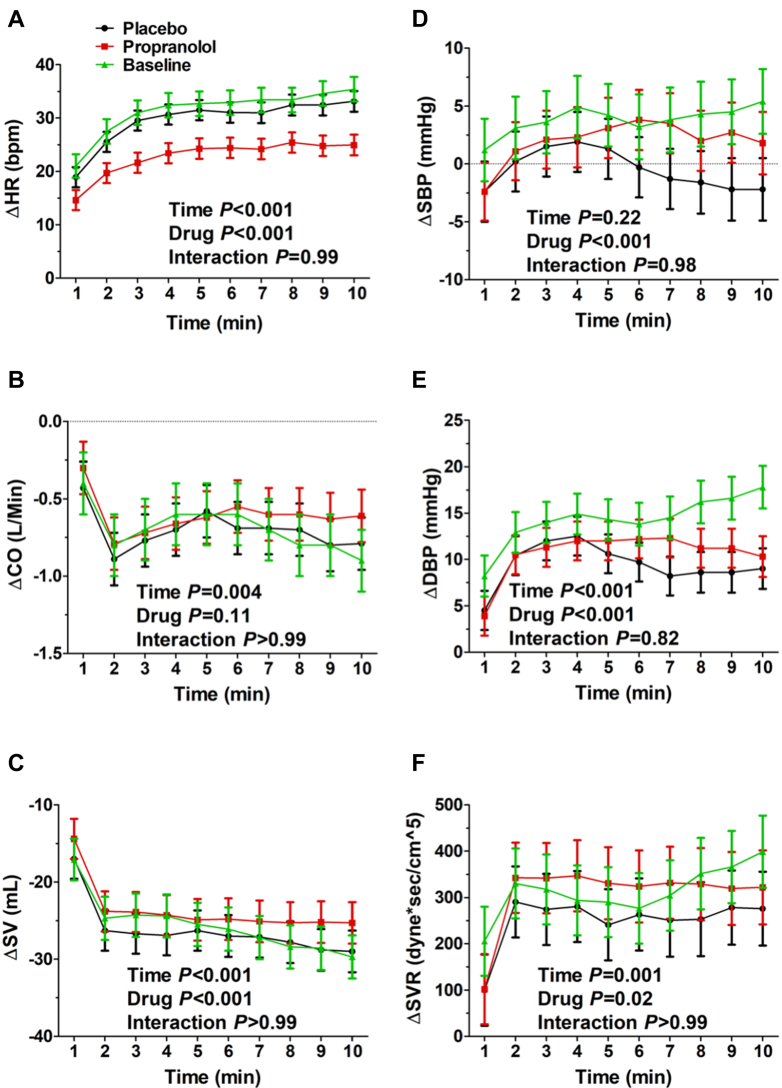
**Delta Hemodynamics (Propranolol vs Placebo) Delta Hemodynamics During Full 10-Minutes of Head-Up-Tilt for Propranolol, Placebo, and Visit 1 (Baseline)** Estimated marginal mean data for delta (A) heart rate, (B) cardiac output, (C) stroke volume, (D) systolic blood pressure, (E) diastolic blood pressure, and (F) systemic vascular resistance are shown across the full 10-minutes of HUT for propranolol (red), placebo (black), and baseline visit (green). Data are shown for n = 28 participants. Data are represented as mean ± SEM. Numbers indicate significance level for time, propranolol vs placebo (drug), and interaction from a linear mixed model. Less than *P* = 0.05 was considered significant. Abbreviations as in Figure 3.

ΔHR increased significantly over time (P_Time_ < 0.001) (Figure 4A). ΔCO exhibited a time-dependent decrease (P_Time_ = 0.004) (Figure 4B). ΔSV exhibited a time-dependent decrease (P_Time_ <0.001) and had a less magnitude decrease with propranolol (−26 mL vs −24 mL; P_Drug_<0.001) (Figure 4C). ΔSBP experienced an increase with propranolol (−0.2 mm Hg vs 2.2 mm Hg; P_Drug_ <0.001) (Figure 4D). ΔDBP increased over time (P_Time_ <0.001) and was larger in magnitude for propranolol (9.4 mm Hg vs 10.9 mm Hg; P_Drug_<0.001) (Figure 4E). Lastly, ΔSVR increased significantly over time (P_Time_ = 0.001) and was larger in magnitude for propranolol (247 dyn·s·cm^-5^ vs 309 dyn·s·cm^-5^; P_Drug_ = 0.02) (Figure 4F). None of the delta hemodynamics displayed interaction effects. There was a greater OI with propranolol (4.9 ± 0.6 vs 4.2 ± 0.5; *P* = 0.006) compared to placebo. Absolute minute-by-minute data for each drug are provided in Supplemental Figure 2.

#### Peak HR hemodynamics during HUT

Pertinent positives at HR_Peak_ can be found in the concurrent JACC brief report.

#### 24-hour Bittium Holter monitor

The day and night mean HR was lower for propranolol compared to placebo (Table 3). There was a lower maximum HR recorded during night for propranolol, but no differences in maximum HR over 24-hours and during the day (Table 3).

#### Participant reported outcomes

Propranolol demonstrated lower post-tilt mental clouding (4.9 ± 3.1 vs 3.6 ± 2.9; *P* = 0.005), rapid heartbeat (5.0 ± 2.7 vs 3.8 ± 2.8; *P* = 0.01), and overall score (38.8 ± 21.9 vs 32.4 ± 22.8; *P* = 0.04) compared to placebo (Table 4).

Propranolol demonstrated less muscle ache for MAPS compared to placebo (4.7 ± 3.0 vs 3.7 ± 3.0; *P* = 0.01) (Table 4).

There were no differences in baseline VOSS, ΔVOSS, or Anxiety VAS between propranolol and placebo (Table 4).

### Ivabradine vs propranolol

#### Hemodynamic response to HUT

**Figure 5:**
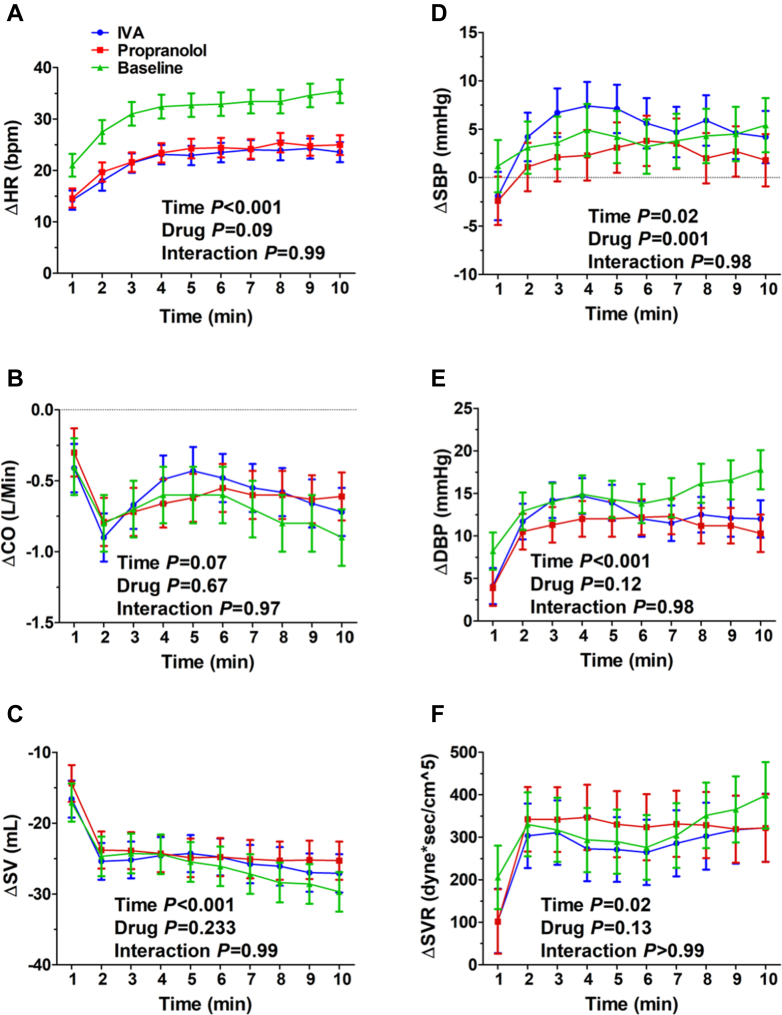
**Delta Hemodynamics (Ivabradine vs Propranolol) Delta Hemodynamics During Full 10-Minutes of Head-Up-Tilt for Ivabradine, Propranolol, and Visit 1 (Baseline)** Estimated marginal mean data for (A) delta heart rate, (B) cardiac output, (C) stroke volume, (D) systolic blood pressure, (E) diastolic blood pressure, and (F) systemic vascular resistance are shown across the full 10-minutes of HUT for ivabradine (blue), propranolol (red), and baseline visit (green). Data are shown for n = 28 participants. Data are represented as mean ± SEM. Numbers indicate significance level for time, ivabradine vs propranolol (drug), and interaction from a linear mixed model. Less than *P* = 0.05 was considered significant. Abbreviations as in Figure 3.

ΔHR increased significantly over time (P_Time_<0.001) and showed a nonsignificant time × drug interaction, indicating that the trajectory of change over time did not differ between ivabradine and propranolol (P_Int_ = 0.99) (Figure 5A). ΔCO showed no differences in time, drug, or interaction effects (P_Time_ = 0.07; P_Drug_ = 0.67; P_Int_ = 0.97) (Figure 5B). ΔSV exhibited a time-dependent decrease (P_Time_ <0.001) (Figure 5C). ΔDBP increased over time (P_Time_ <0.001) (Figure 5E). Lastly, ΔSVR increased significantly over time (P_Time_ = 0.02) (Figure 5F). None of the delta hemodynamics displayed interaction effects. There was no difference in OI between ivabradine and propranolol (5.2 ± 0.5 vs 4.9 ± 0.6; *P*= 0.39). Absolute minute-by-minute data for each drug are provided in Supplemental Figure 3.

#### Peak HR during HUT

Aside from HR_Peak_ and ΔHR_Peak_, there were no other differences in the absolute or delta hemodynamics at HR_Peak_.

#### 24-hour Bittium Holter monitor

There were no differences in day and night mean HR between ivabradine and propranolol (Table 3). There were no differences in maximum HR recorded over day or night (Table 3).

#### Participant-reported outcomes

Propranolol showed a greater magnitude of shortness of breath compared to ivabradine during baseline VOSS (1.3 ± 1.5 vs 0.8 ± 1.0; *P* = 0.01) (Table 4).

There were no differences in post-tilt VOSS, ΔVOSS, MAPS, and anxiety VAS between ivabradine and propranolol.

### Order effect analysis

Using ΔHR following HUT, there were no significant differences in ΔHR across phases 1, 2, and 3 (28 ± 2 beats/min vs 31 ± 2 beats/min vs 31 ± 2 beats/min; *P* = 0.20), indicating no evidence of an order/period effect.

### Cognitive assessment

No other cognitive domains aside from reaction time differ between treatment phases.

### Qualitative interviews

Eleven individuals completed the qualitative interviews. One subgroup, 73% (8/11), preferred ivabradine over propranolol due to better HR control when standing, reduction in symptom frequency and intensity, and reduced fatigue. One participant noted that propranolol made her feel heavy, like “trudging through mud”. Another participant noted she could feel propranolol wear off more abruptly, whereas ivabradine faded away slowly, allowing more accurate timing of her next dose. Another participant noted that propranolol was “awful” as it reduced her blood pressure, induced more syncope episodes, and worsened symptom severity. One participant expressed a desire to switch to ivabradine but remained on propranolol because her insurance did not cover ivabradine.

Of those that favored propranolol (3/11; 27%), 2 participants noted that propranolol reduced their symptoms more than ivabradine. The last participant noted that when they tried ivabradine, they noticed no changes except for visual symptoms which made her uncomfortable.

### Hyperadrenergic vs nonhyperadrenergic POTS

HR differences between participants with and without hyperadrenergic POTS for either ivabradine or propranolol can be found in the concurrent *JACC* Brief Report. The full comparison is provided in Supplemental Table 1.

## Discussion

We conducted a double-blinded, placebo-controlled, randomized crossover trial of ivabradine, propranolol, and placebo in patients with POTS.^28^ The findings of this study highlight important distinctions and similarities in the hemodynamic responses, symptom management, and participant-reported outcomes between ivabradine and propranolol. Our findings demonstrated that both ivabradine and propranolol were similar at reducing HR but demonstrated differences in blood pressure. Both were able to significantly reduce HR compared to placebo. Symptoms were similar for both active drugs, but some scores improved with the active drugs compared to placebo. Finally, although there were no major differences in symptom management between the active drugs, there was a divide in patient preferences, neither clearly favoring ivabradine or propranolol. To summarize, although both active treatments demonstrated improvements compared with placebo, direct comparisons revealed few significant differences between ivabradine and propranolol across patient-reported, functional, and cognitive outcomes. Overall, the separation was more pronounced between active therapy and placebo than between the 2 active medications. These results provide the first direct, randomized comparative evidence that ivabradine and propranolol produce overlapping short-term hemodynamic and symptomatic improvements in POTS, with no statistically significant differences detected between active treatments.

### Prior studies and beneficial usage of ivabradine and beta-blockers in POTS

There have been smaller trials of ivabradine and other beta-blockers in POTS. One randomized placebo-controlled trial examining ivabradine demonstrated a significant reduction in HR compared to placebo, similar to the results discussed here.^4^ All patients were of the “hyperadrenergic subtype” and had elevated baseline norepinephrine levels. Ivabradine was able to lower norepinephrine levels compared to placebo. This study did not compare hyperadrenergic POTS to nonhyperadrenergic POTS. Beta-blockers, specifically propranolol, have been shown, in low doses, to improve orthostatic tachycardia and reduce norepinephrine levels in these patients.^6^ Although prior studies have suggested greater efficacy of ivabradine in hyperadrenergic POTS, we did not observe differences in HR_Peak_ or ΔHR_Peak_ between hyperadrenergic and nonhyperadrenergic participants for either ivabradine or propranolol.

The recent Canadian Cardiovascular Society position statement on POTS highlighted that patients with low supine blood pressure should be prescribed midodrine or pyridostigmine.^3^ We found that ivabradine elevated SBP more than propranolol. Perhaps ivabradine could be used in cases of low supine blood pressure.

### Ivabradine and propranolol improve orthostatic tachycardia without compromising other hemodynamics

Both ivabradine and propranolol significantly reduced the orthostatic HR increase compared to placebo, consistent with earlier studies.^4^^,^^6^^,^^28^ The HR_Peak_ during HUT was also lower with both drugs, reinforcing their ability to blunt excessive orthostatic tachycardia, the key physiological impact of POTS.^2^^,^^3^ This is also seen with our 24-hour HR monitor, where both drugs reduced the mean 24-hour HR compared to placebo, providing real world relevance outside of structured testing.

It is important to mention that ivabradine increased SBP compared to propranolol. This is relevant in POTS as many patients with POTS have low baseline blood pressures, possibly due to reduced blood volume in these patients.^29^ This allows clinicians to adopt a more personalized approach to drug selection.

Our study also demonstrated that both CO and SV decreased as expected with a transition to upright posture,^30^ with no differences between the drugs. Clinically, HR-lowering medications have been concerning, particularly beta-blockers, which could worsen symptoms by reducing CO in patients who rely on tachycardia to maintain proper systemic function.^6^^,^^31^ Our findings suggest that HR-lowering drugs do not impair the compensatory mechanisms essential for upright tolerance in POTS.

### Improvement in orthostatic symptoms but limited impact on global QoL

Both medications improved orthostatic symptoms relative to placebo including shortness of breath, rapid heartbeat, mental clouding, and overall orthostatic symptom scores. These improvements align with prior evidence that reducing HR can alleviate acute symptoms in POTS.^6^^,^^9^

Importantly, no significant between-drug differences were observed in the majority of symptom or QoL measures, including RAND-36, MAPS, and anxiety VAS scores. This difference between improved orthostatic symptoms and unchanged QoL underscores the multidimensional nature of POTS, where symptoms such as fatigue, cognitive dysfunction, sleep disturbance, pain, and gastrointestinal symptoms contribute to overall disease burden.^3^ Although HR-lowering reduces cardinal orthostatic symptoms, it alone may be insufficient to improve broader QoL domains. Cognitive dysfunction is a prominent and disabling feature of POTS. In this study, propranolol improved standing reaction time compared with placebo, suggesting a potential benefit on upright psychomotor performance. Although no other cognitive domains differed between treatments, this finding highlights a possible interaction between HR control and cognitive function that warrants further investigation. This potentially emphasizes the need for multimodal treatment approaches, such as nonpharmacological interventions which include increased salt/water intake, exercise, and compression therapy.^3^ In addition, a longer time on medication may be needed to observe differences in these QoL measures.

### Ivabradine and propranolol show some physiological equivalence but divergent participant experience

Despite distinct methods of action, both ivabradine and propranolol produced similar hemodynamic effects. There were no differences in HR_Peak_ between the 2 drugs. This similarity suggests that at doses tested, both drugs elicit similar degrees of orthostatic HR reduction (seen via HUT) and sinus HR reduction (seen via 24-hour Holter monitor). Importantly, both ivabradine and propranolol demonstrated a ΔHR_Peak_ that was below diagnostic threshold for POTS (≥30 beats/min), indicating meaningful improvement but no major differences between the active drugs.

Despite physiologic equivalence at the group level, individual participants demonstrated clear preferences for one medication over the other. Although overall group preferences did not differ statistically between ivabradine and propranolol, the distribution was divided, suggesting meaningful interindividual variability in perceived benefit and tolerability. Qualitative interviews demonstrated that participants reported greater symptom intensity, fatigue, and abrupt wearing-off effects with propranolol, whereas ivabradine offered smoother HR control and better daily energy levels. These findings indicate an important point in POTS care: patient-centered outcomes may diverge from objective physiologic metrics. Even when 2 medications provide equivalent hemodynamic benefit at the population level, differences in blood pressure response, tolerability, and patient experience may guide individualized therapy. This is especially important for POTS as QoL is often impaired.

In addition, it is important to note that both ivabradine and propranolol are now generic medications; however, a cost-discrepancy between these medications still does exist and may limit access to ivabradine (Central Illustration).

**Central illustration:**
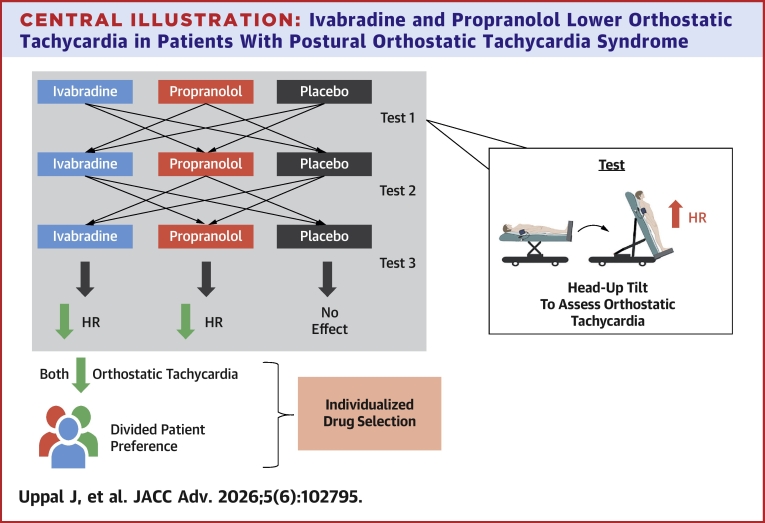
**Ivabradine and Propranolol Lower Orthostatic Tachycardia in Patients With Postural Orthostatic Tachycardia Syndrome** HR = heart rate.

### Strengths, limitations, and future directions

This study has several strengths: it is the largest randomized, double-blind, placebo-controlled, crossover drug trial directly comparing ivabradine and propranolol in POTS and builds on prior studies examining the 2 drugs separately. In addition, there was 24-hour Holter monitoring for real world relevance, along with multiple validated symptoms and QoL surveys. Integration with a qualitative patient-experience study further strengthens the patient centered relevance of findings.

Some limitations include limited assessment of long-term efficacy or tolerance due to the 4-week drug duration in accordance with the crossover design; however, this design enhances statistical power. The MAPS survey may have been underpowered due to it being integrated into the study late (n = 14). The cohort was all female, potentially limiting generalizability, but this is consistent with POTS epidemiology.^3^ There may have been residual carryover effects, although this was mitigated by appropriate washout periods in between-drug phases. Finally, the study did have a high dropout rate, but due to the larger delta HR changes observed, the study still met statistical power.

## Conclusions

Both ivabradine and propranolol improved orthostatic tachycardia and symptoms in patients with POTS compared to placebo. Ivabradine demonstrated a greater increase in SBP compared to propranolol, which could be clinically meaningful. The 2 medications produced largely comparable hemodynamic and symptomatic responses, with no statistically significant differences detected in most measures, specifically HR reduction and overall symptom scores. Notably, there was a trend to a preference for ivabradine, due to better tolerability and smoother HR control, but this was not statistically significant. These findings support both drugs as effective options for managing POTS, whereas underscoring the need for individualized therapy and future studies evaluating long-term and multimodal treatment strategies.

**What is the clinical question being addressed?**

Does ivabradine or propranolol more effectively reduce orthostatic tachycardia and symptoms in postural orthostatic tachycardia syndrome?

**What is the main finding?**

Both ivabradine and propranolol reduce tachycardia and symptoms in postural orthostatic tachycardia syndrome, supporting their use, but variability in response suggests individualized treatment selection is warranted.

Perspectives **COMPETENCY IN PATIENT CARE:** In adults with POTS, both ivabradine and propranolol effectively reduce orthostatic tachycardia compared to placebo, with similar hemodynamic effects. These findings support the use of HR lowering pharmacotherapy as a treatment option in POTS. Selection of medication should be guided by patient tolerance and clinical response.

**TRANSLATIONAL OUTLOOK:** Future studies should identify physiological and clinical predictors of differential response to ivabradine and propranolol. In addition, longer-term outcomes to inform individualized, mechanism-based treatment strategies are needed for patients with POTS.

## Funding support and author disclosures

This study was partially funded by Dysautonomia International (East Moriches, NY, USA), by the Libin Cardiovascular Institute (Calgary, AB, Canada), and by the 10.13039/100006108National Center for Advancing Translational Sciences Award UL1 TR000445. Dr Raj is a consultant to Regeneron, argenx BV, Antag Pharma, and Lumia Health; and he received grants from 10.13039/100004411Heart & Stroke Foundation of Canada, Dysautonomia International, Standing Up To POTS, and the Canerector Foundation. All other authors have reported that they have no relationships relevant to the contents of this paper to disclose.

## Supplementary material

Supplementary docx 1
