# Supplementary material for: A Randomized Crossover Trial of Ivabradine, Propranolol, and Placebo in Postural Orthostatic Tachycardia Syndrome: A Detailed Description
Source: JACC Adv. 2026 May 13;5(6):102795. doi: 10.1016/j.jacadv.2026.102795 (PMC13308246; doi:10.1016/j.jacadv.2026.102795)
Supplement: Supplementary docx 1 [file mmc1.docx]

**Online Figure 1.** Absolute Hemodynamics (IVA vs. Placebo)


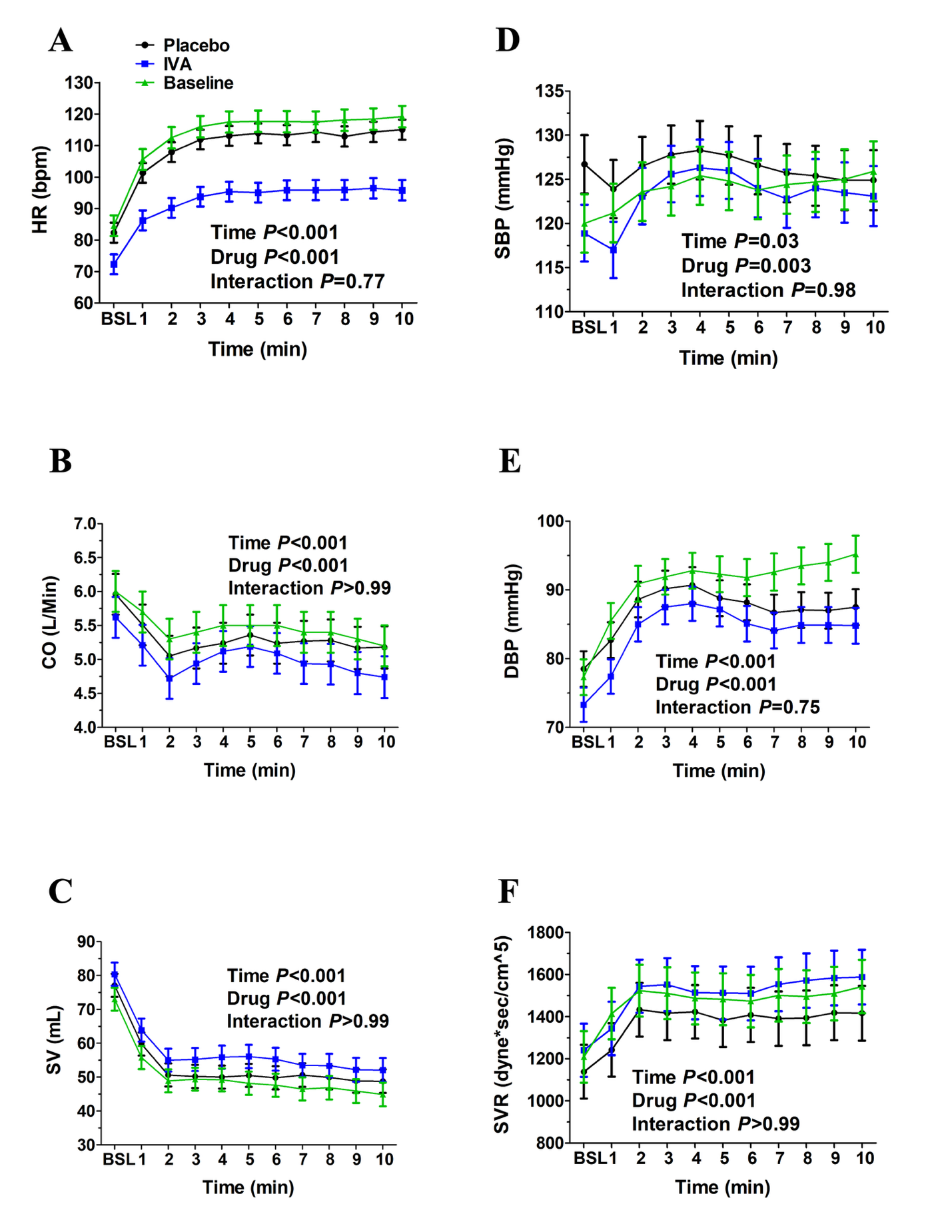


**Online Figure 2.** Absolute Hemodynamics (Propranolol vs. Placebo)


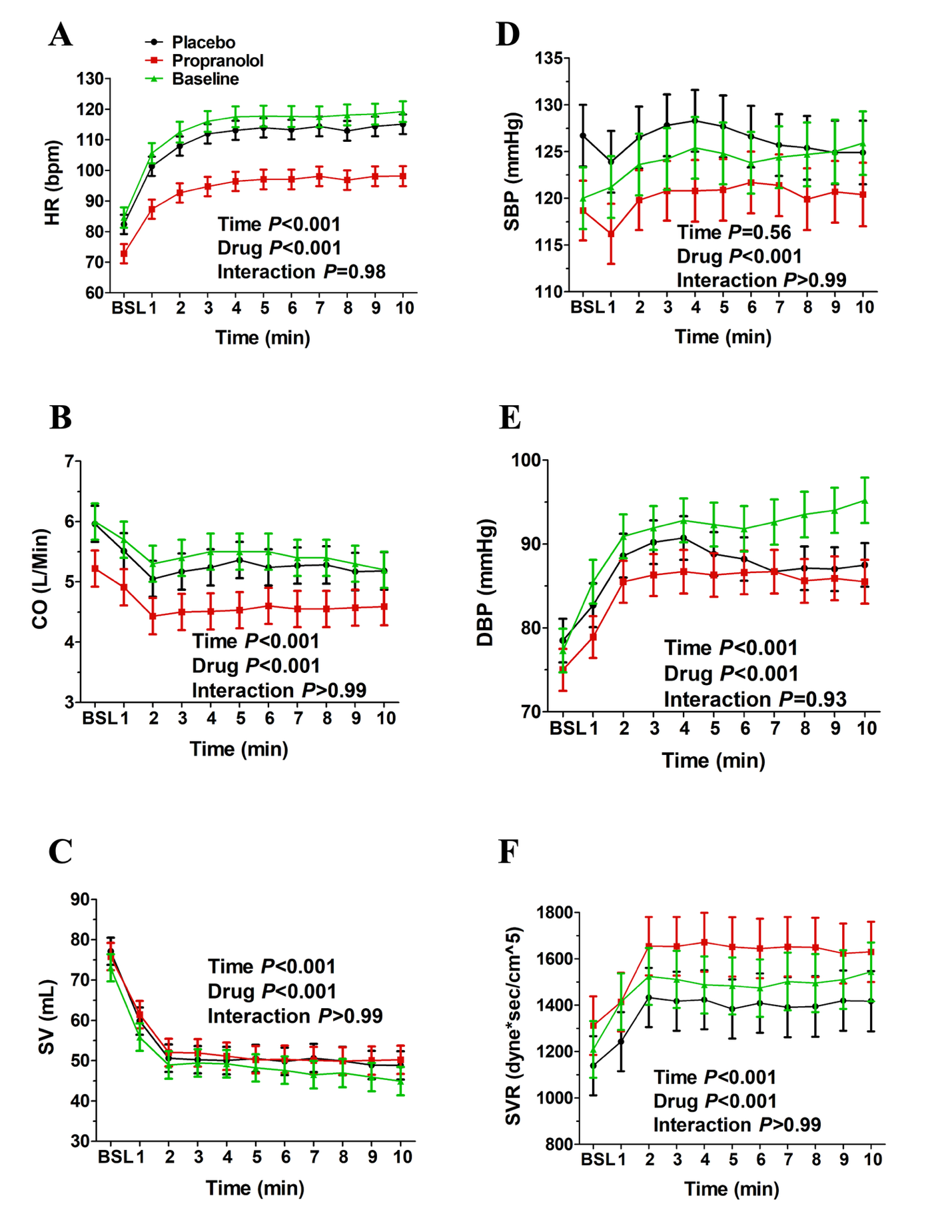


**Online Figure 3.** Absolute Hemodynamics (IVA vs. Propranolol)


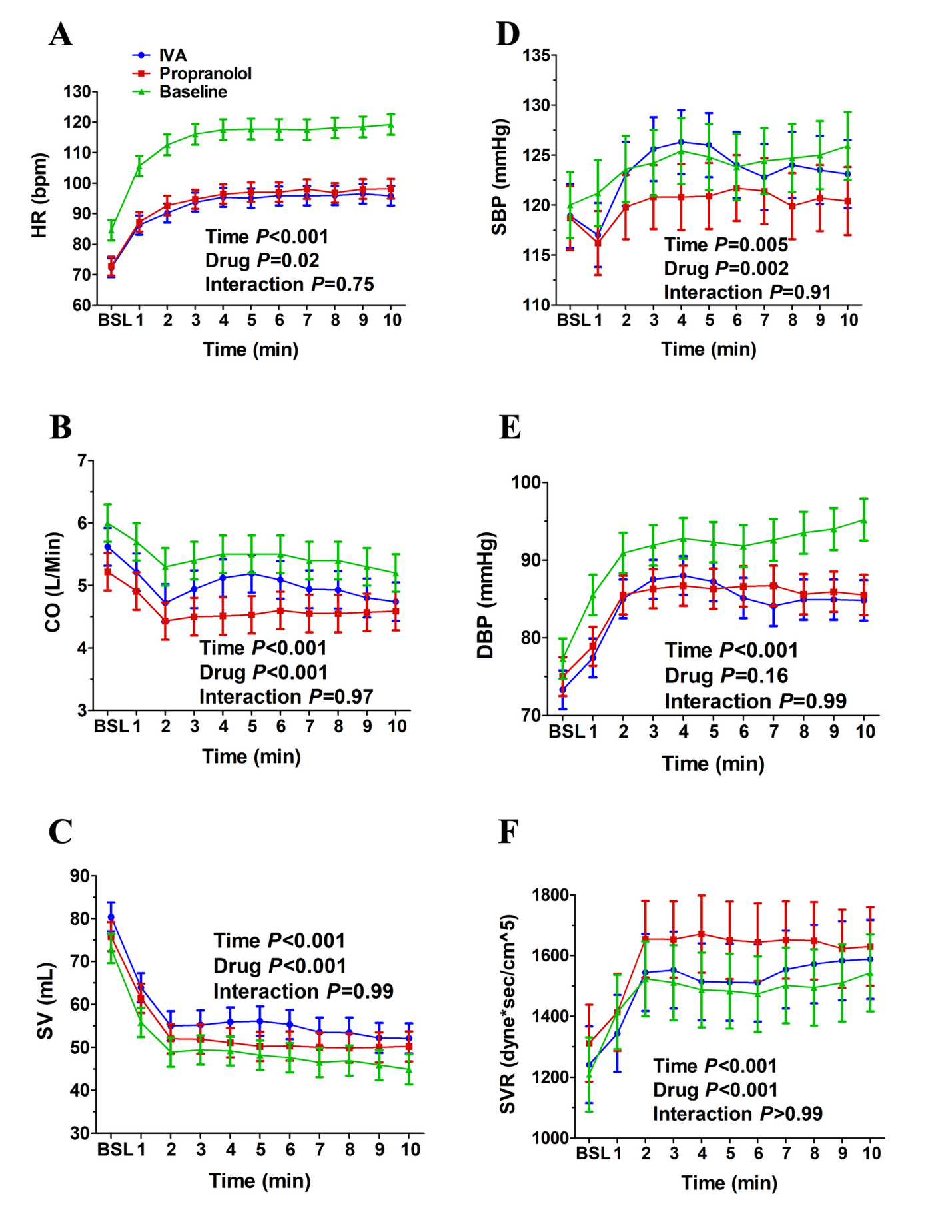


| Outcome | Ivabradine | | | Propranolol | | |
| --- | --- | --- | --- | --- | --- | --- |
| Variable | **Hyperadrenergic POTS (n=11)** | **Non-Hyperadrenergic (n=6)** | **p-value** | **Hyperadrenergic POTS (n=9)** | **Non-Hyperadrenergic (n=6)** | **p-value** |
| Absolute Peak HR (bpm) | 100±20 | 95±9 | 0.34 | 103±22 | 101±15 | 0.38 |
| Δ Peak HR (bpm) | 27±13 | 27±8 | 0.92 | 32±11 | 29±8 | 0.48 |

**Online Table 1.** Heart Rate Assessment of Hyperadrenergic POTS versus Non-Hyperadrenergic POTS

**Explanatory Notes:** Participants were stratified based on hyperadrenergic status, defined by upright norepinephrine levels (≥600 pg/mL). Absolute peak heart rate (HR) and delta (Δ) peak HR were assessed during head-up tilt testing. Δ peak HR was calculated as the difference between peak HR during tilt and supine baseline HR. Data are presented as mean ± standard deviation. P-values represent comparisons between hyperadrenergic and non-hyperadrenergic groups within each drug condition using a Mann–Whitney U test. A p-value < 0.05 was considered statistically significant. HR, heart rate; POTS, Postural Orthostatic Tachycardia Syndrome.

**ONLINE FIGURE LEGENDS**

**Online Figure 1. Absolute Hemodynamics (IVA vs. Placebo)**

Absolute hemodynamics during full 10-minutes of HUT for ivabradine, placebo, and visit 1 (baseline). Estimated marginal mean data for heart rate (A), cardiac output (B), stroke volume (C), systolic blood pressure (D), diastolic blood pressure (E), and systemic vascular resistance (F) are shown across the full 10-minutes of HUT for ivabradine (blue), placebo (black), and baseline visit (green). Data are shown for n=28 participants. Data are represented as mean ± standard error of the mean. Numbers indicate significance level for time, ivabradine vs. placebo (drug), and interaction from a linear mixed model. Less than *p* = 0.05 was considered significant. HUT, Head-up tilt.

**Online Figure 2. Absolute Hemodynamics (Propranolol vs. Placebo)**

Absolute hemodynamics during full 10-minutes of HUT for propranolol, placebo, and visit 1 (baseline). Estimated marginal mean data for heart rate (A), cardiac output (B), stroke volume (C), systolic blood pressure (D), diastolic blood pressure (E), and systemic vascular resistance (F) are shown across the full 10-minutes of HUT for propranolol (red), placebo (black), and baseline visit (green). Data are shown for n=28 participants. Data are represented as mean ± standard error of the mean. Numbers indicate significance level for time, propranolol vs. placebo (drug), and interaction from a linear mixed model. Less than *p* = 0.05 was considered significant. HUT, Head-up tilt.

**Online Figure 3. Absolute Hemodynamics (IVA vs. Propranolol)**

Absolute hemodynamics during full 10-minutes of HUT for ivabradine, propranolol, and visit 1 (baseline). Estimated marginal mean data for heart rate (A), cardiac output (B), stroke volume (C), systolic blood pressure (D), diastolic blood pressure (E), and systemic vascular resistance (F) are shown across the full 10-minutes of HUT for ivabradine (blue), propranolol (red), and baseline visit (green). Data are shown for n=28 participants. Data are represented as mean ± standard error of the mean. Numbers indicate significance level for time, ivabradine vs. propranolol (drug), and interaction from a linear mixed model. Less than *p* = 0.05 was considered significant. HUT, Head-up tilt.
